# Therapeutic Response After 10 Months of Treatment With Letrozole and Palbociclib in a Postmenopausal Woman With Hormone Receptor-Positive (HR+), Human Epidermal Growth Factor Receptor 2-Negative (HER2-) Metastatic Breast Cancer and Skin Involvement

**DOI:** 10.7759/cureus.79174

**Published:** 2025-02-17

**Authors:** Ivan Bivolarski

**Affiliations:** 1 Medical Oncology, Integrated Oncology Centre, Burgas, BGR

**Keywords:** cdk4/6 inhibitors, denosumab, hormonal therapy, hormone receptor-positive (hr+), human epidermal growth factor receptor 2-negative (her2-)- breast cancer, letrozol, metastatic breast cancer (mbc), pain management, palbociclib, skin involvement

## Abstract

Metastatic breast cancer (mBC) remains a major therapeutic challenge, particularly in postmenopausal women with hormone receptor-positive (HR+), human epidermal growth factor receptor 2-negative (HER2-) disease. The introduction of CDK4/6 inhibitors in combination with endocrine therapy has significantly improved outcomes in these patients. We present the case of a postmenopausal woman diagnosed with HR+, HER2-negative mBC with cutaneous involvement, who was initiated on letrozole and palbociclib as first-line therapy. After ten months of treatment, she demonstrated a remarkable therapeutic response, with significant clinical and radiological improvements. This case highlights the potential benefits of CDK4/6 inhibitors combined with endocrine therapy in the management of HR+, HER2-negative metastatic breast cancer, particularly in cases with skin involvement. These agents have reshaped the therapeutic landscape, providing prolonged disease control and improved patient outcomes.

## Introduction

Hormone receptor-positive (HR+), human epidermal growth factor receptor 2-negative (HER2-) breast cancer is the most common molecular subtype, accounting for 60-70% of cases [1]. Despite significant advancements in treatment, the optimal therapeutic sequence and the impact of combining bone-modifying agents with targeted therapies remain areas of active investigation.

Patients with HR+, HER2- metastatic breast cancer (mBC), and cutaneous involvement often face poor prognosis and significantly reduced quality of life due to refractory disease and limited treatment options. This case highlights the successful long-term disease control achieved with the combination of letrozole, palbociclib, and denosumab, reinforcing the role of CDK4/6 inhibitors in challenging clinical scenarios. While these inhibitors have become the standard of care, real-world data on their efficacy in patients with cutaneous metastases and concurrent bone-modifying therapy remain limited.

Before the introduction of cyclin-dependent kinase 4/6 (CDK4/6) inhibitors, treatment for HR+, HER2- mBC relied solely on hormonal therapy, often leading to disease progression or relapse. The advent of CDK4/6 inhibitors revolutionized the therapeutic landscape, significantly extending progression-free survival (PFS) and overall survival (OS) [2].

Palbociclib (Ibrance), the first approved CDK4/6 inhibitor, received European Medicines Agency authorization in 2016, based on the PALOMA trials, which demonstrated that the combination of palbociclib and endocrine therapy significantly improved PFS compared to endocrine therapy alone [3]. Similar benefits have been observed with ribociclib and abemaciclib, as confirmed by the MONALEESA and MONARCH trials [4,5].

Updated results from the PALOMA-2 trial (38-month follow-up) confirmed that adding palbociclib to letrozole nearly doubled PFS compared to letrozole with placebo (hazard ratio = 0.56; mPFS 27.6 months vs. 14.5 months; p < 0.0001) [3]. These findings align with those from the MONALEESA trials, which reported substantial survival benefits with ribociclib plus endocrine therapy [6].

In this clinical case, the combination of letrozole, palbociclib, and denosumab demonstrated excellent therapeutic outcomes, providing evidence for its efficacy in patients with HR+, HER2- mBC, and cutaneous metastases. Given the aggressive nature of cutaneous involvement, achieving durable disease control in such patients remains a significant clinical challenge. This report adds to the growing body of evidence supporting the integration of bone-targeted agents in combination with CDK4/6 inhibitors, particularly in patients with bone-dominant and cutaneous disease.

## Case presentation

**Figure 1:**
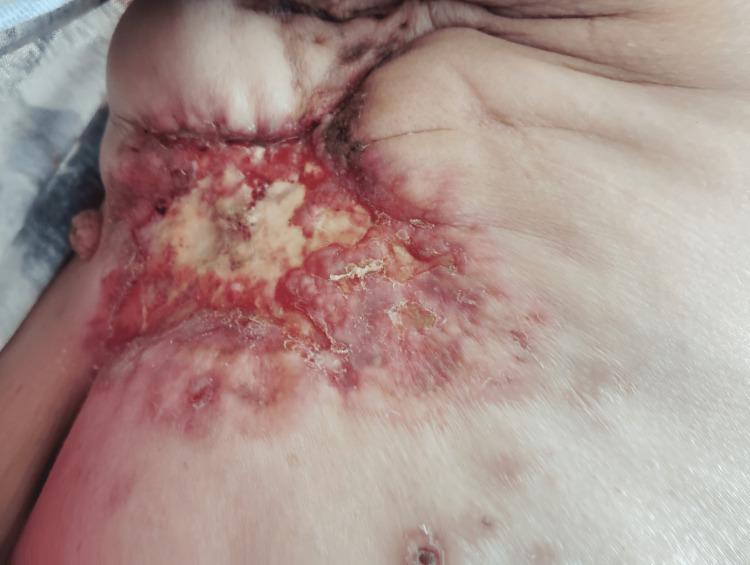
Before treatment

A 58-year-old postmenopausal woman presented to the Integrated Oncology Center (IOC) in Burgas with a non-healing ulcerated wound on her left breast, accompanied by severe pain in the chest, back, and left breast region. The patient had delayed seeking medical attention for months until the pain became unbearable. Upon referral to the Department of Oncosurgery, clinical examination revealed extensive ulceration of the left breast with multiple satellite lesions and palpable axillary lymphadenopathy (Figure 1).

On February 8, 2024, a palliative tumor excision was performed, and histological analysis confirmed invasive ductal carcinoma (G2). Receptor status showed ER(+), PR(+), HER2(1+), and Ki67 at 20%. The initial TNM staging was pT4cN1M0 (stage IIIB).

The patient reported no family history of cancer and had no comorbidities. She had a significant smoking history of over 30 pack-years.

Diagnosis

A comprehensive diagnostic workup included an ultrasound in February 2024, revealing a large, heterogeneous tumor involving most of the breast tissue. A PET/CT scan on February 27, 2024, demonstrated extensive metastatic spread to cervical, supraclavicular, axillary, retroperitoneal, and mediastinal lymph nodes, the right adrenal gland, and bones, confirming stage IV disease (pT4cN1M1).

Treatment and follow-up

**Figure 2:**
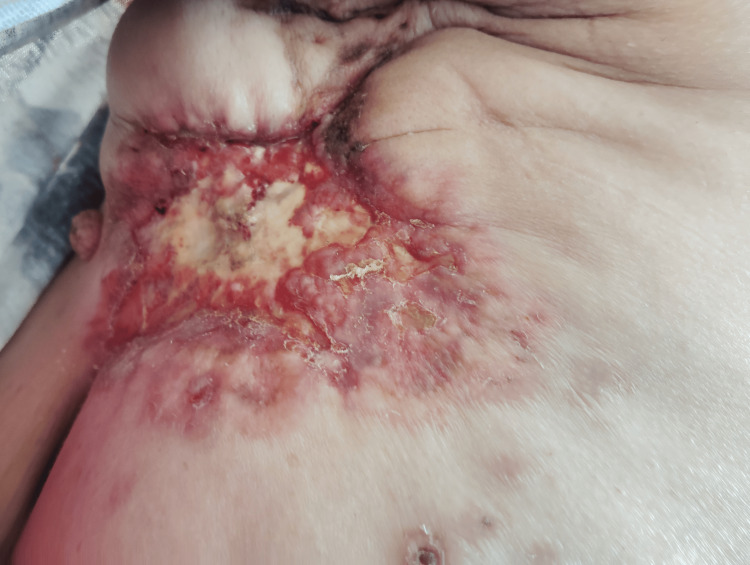
10 days of treatment with letrozole, without the addition of a CDK4/6 inhibitor.

**Figure 3:**
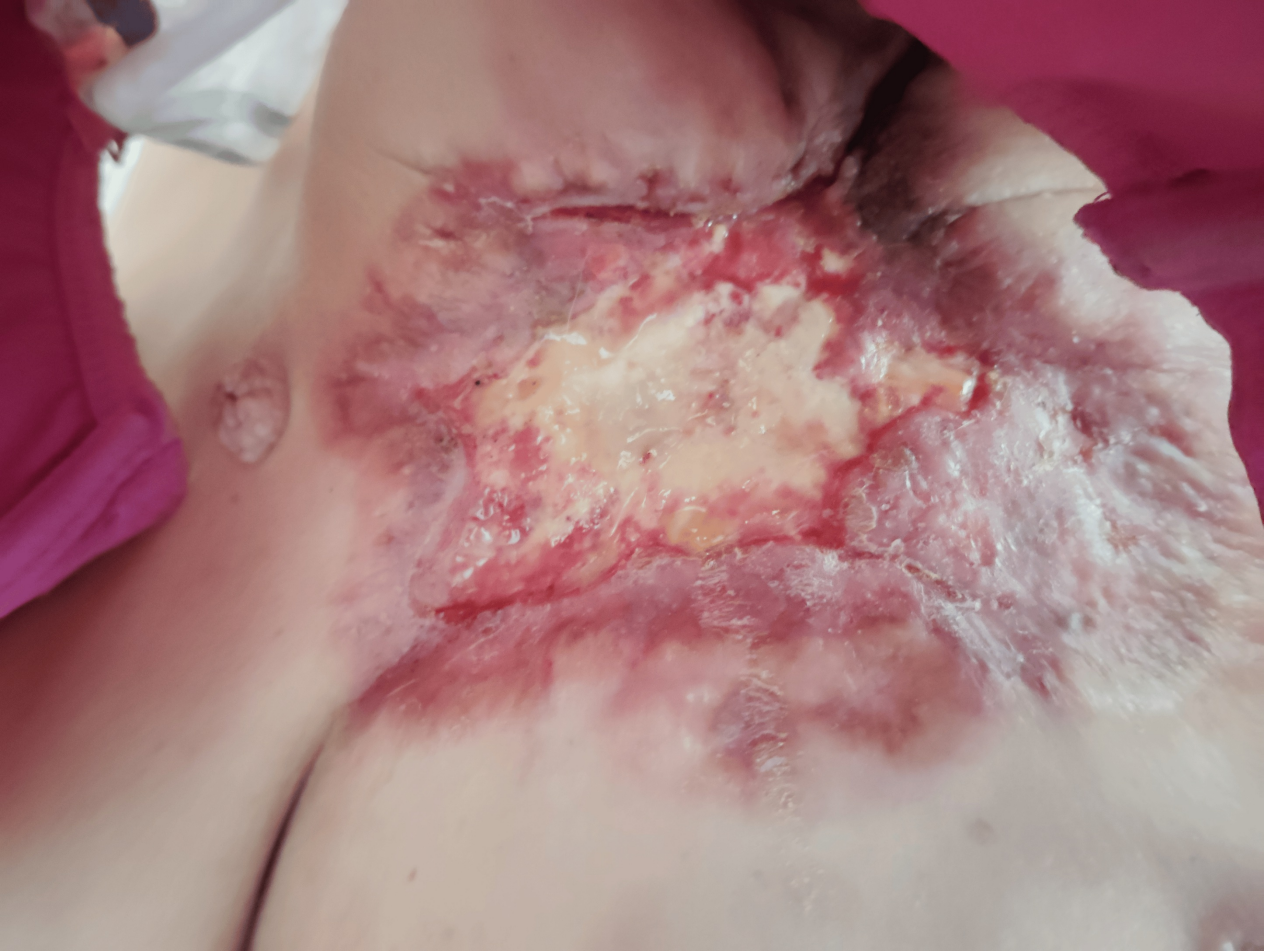
8 days after taking palbociclib

**Figure 4:**
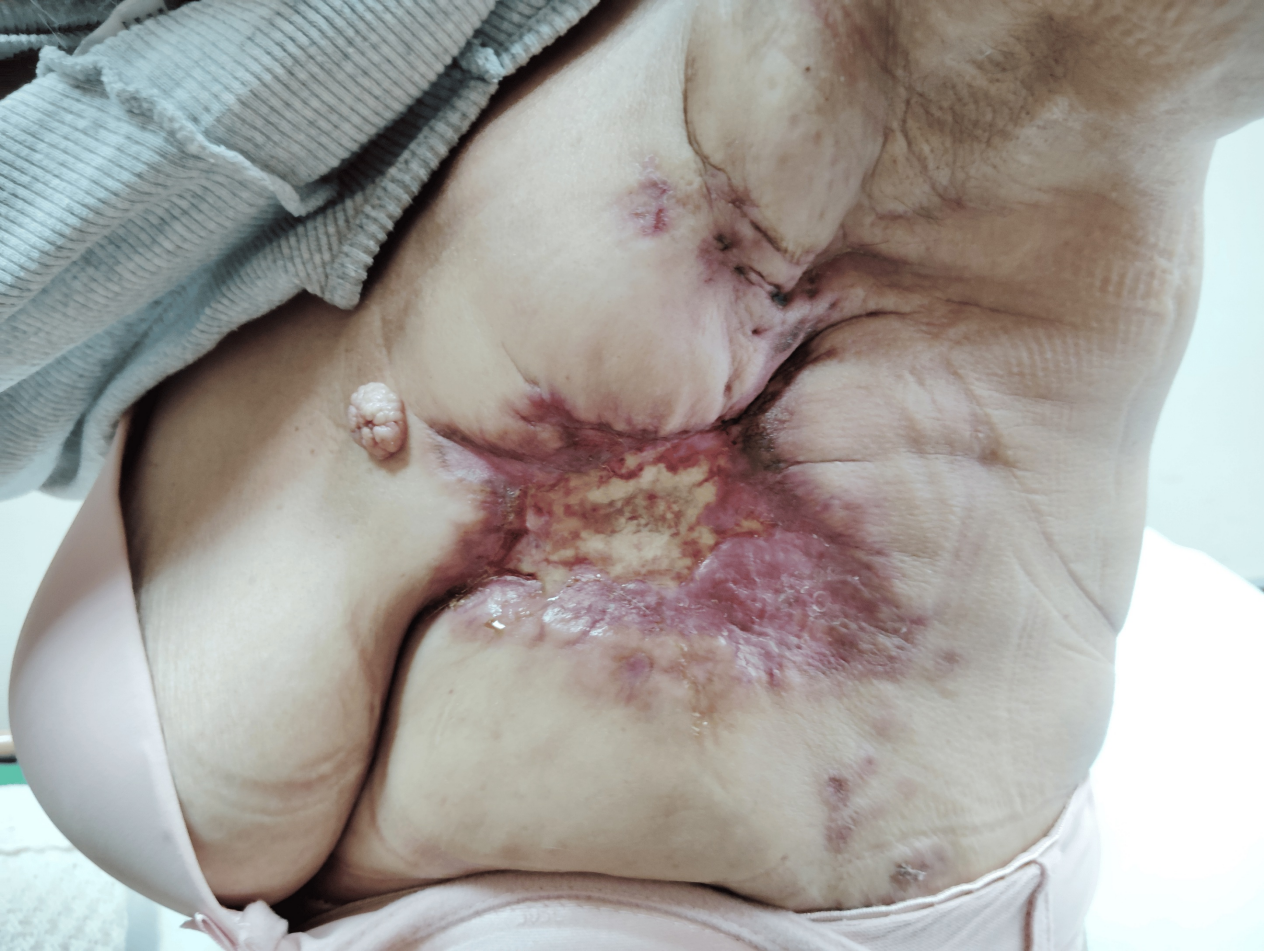
Second cycle of letrozole with palbociclib

On March 22, 2024, the patient initiated hormonal therapy with letrozole (2.5 mg daily) alongside fentanyl for pain management (Figure 2). Due to a short delay in the drug supply process for newly registered patients, palbociclib (125 mg daily, 21 days on, 7 days off) was added on April 3, 2024 (Figure 3). Additionally, denosumab (120 mg every 28 days) was prescribed for bone metastases (Figure 4).

At the start of treatment, the tumor marker cancer antigen (CA 15-3) was 167.25 U/mL (February 5, 2024), with a minimal increase to 167.7 U/mL on April 1, 2024. Following the initiation of palbociclib, a significant decline was observed, with levels dropping to 59.9 U/mL by July 16, 2024, and further decreasing to 43.72 U/mL on November 26, 2024. This biochemical response correlated with the radiological findings, suggesting effective disease control.

**Figure 5:**
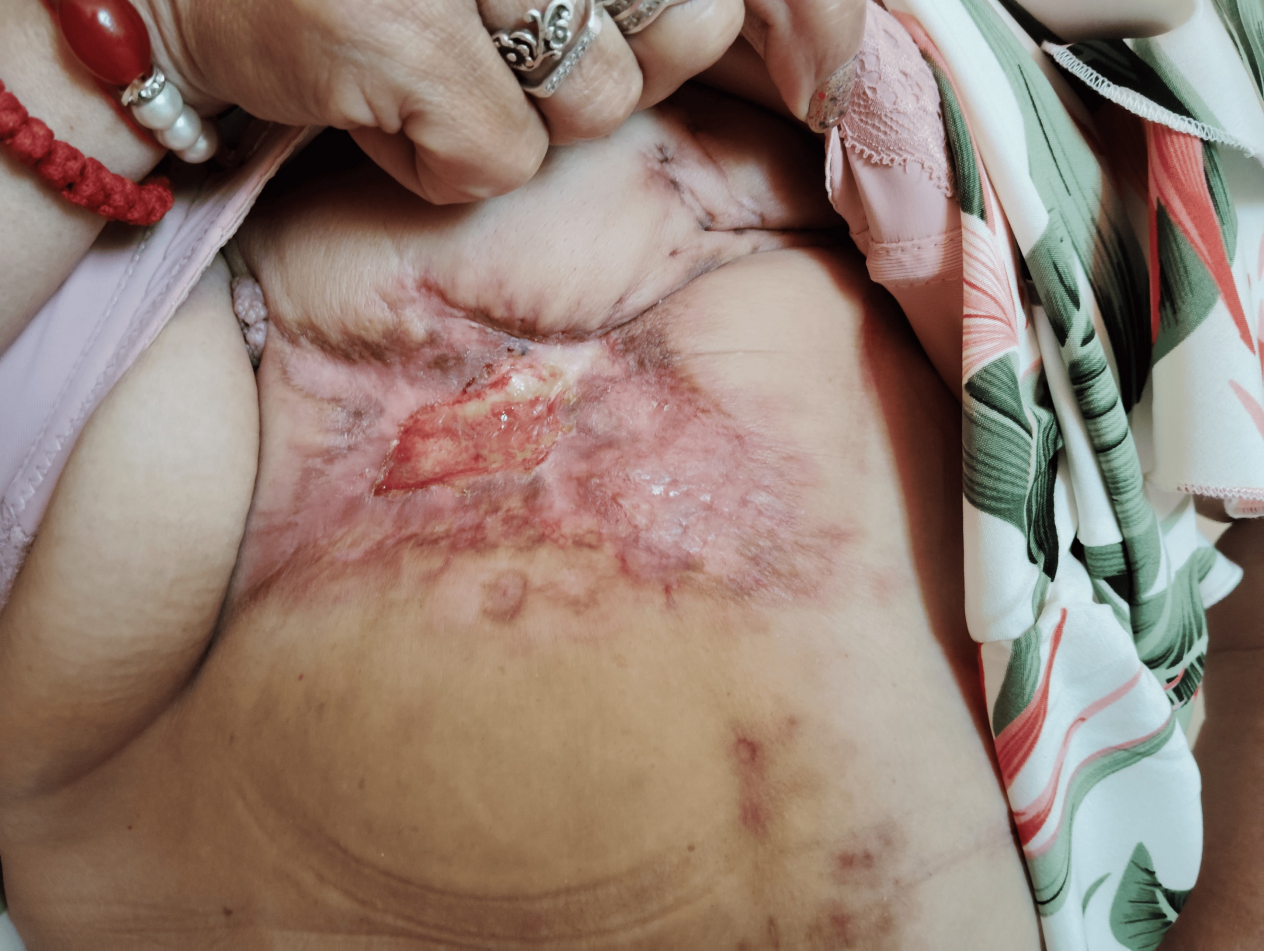
Third cycle of letrozole with palbociclib

**Figure 6:**
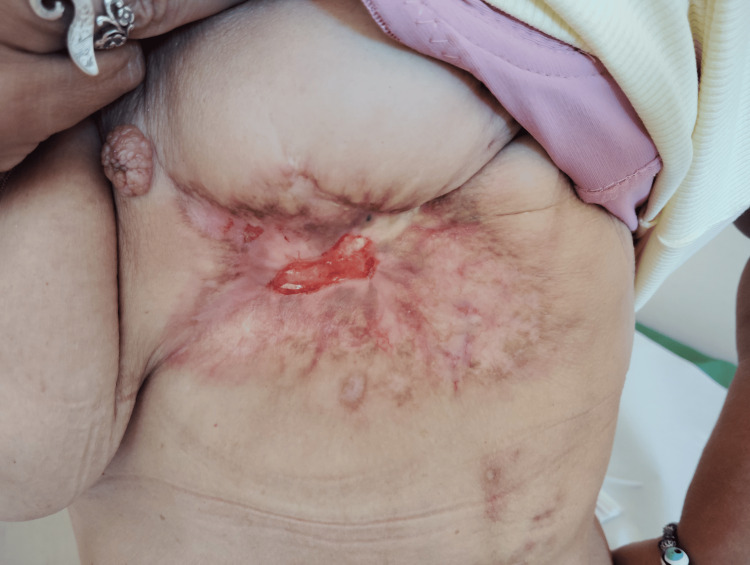
Forth cycle of letrozole with palbociclib

**Figure 7:**
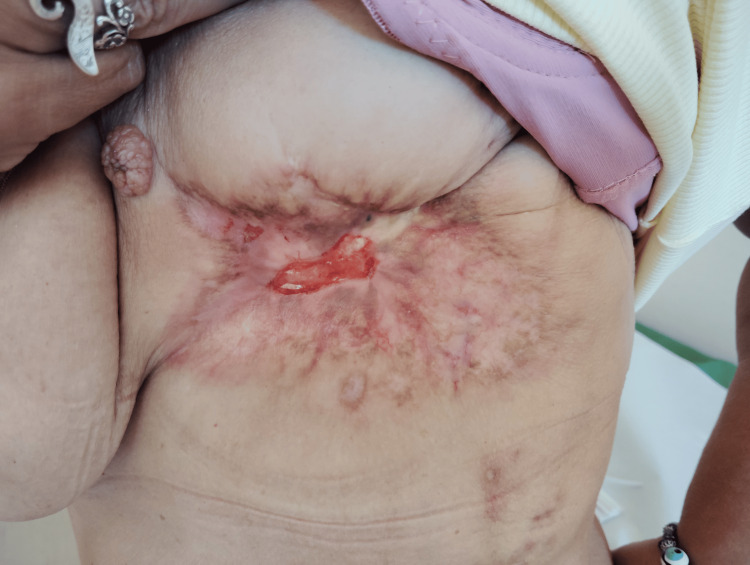
Fifth cycle of letrozole with palbociclib

The treatment plan followed international guidelines recommending CDK4/6 inhibitors in combination with hormonal therapy for HR+, HER2-mBC (Figure 5). This approach was consistent with the PALOMA-2 trial results, emphasizing the efficacy of palbociclib and letrozole (Figures 6-7).

**Figure 8:**
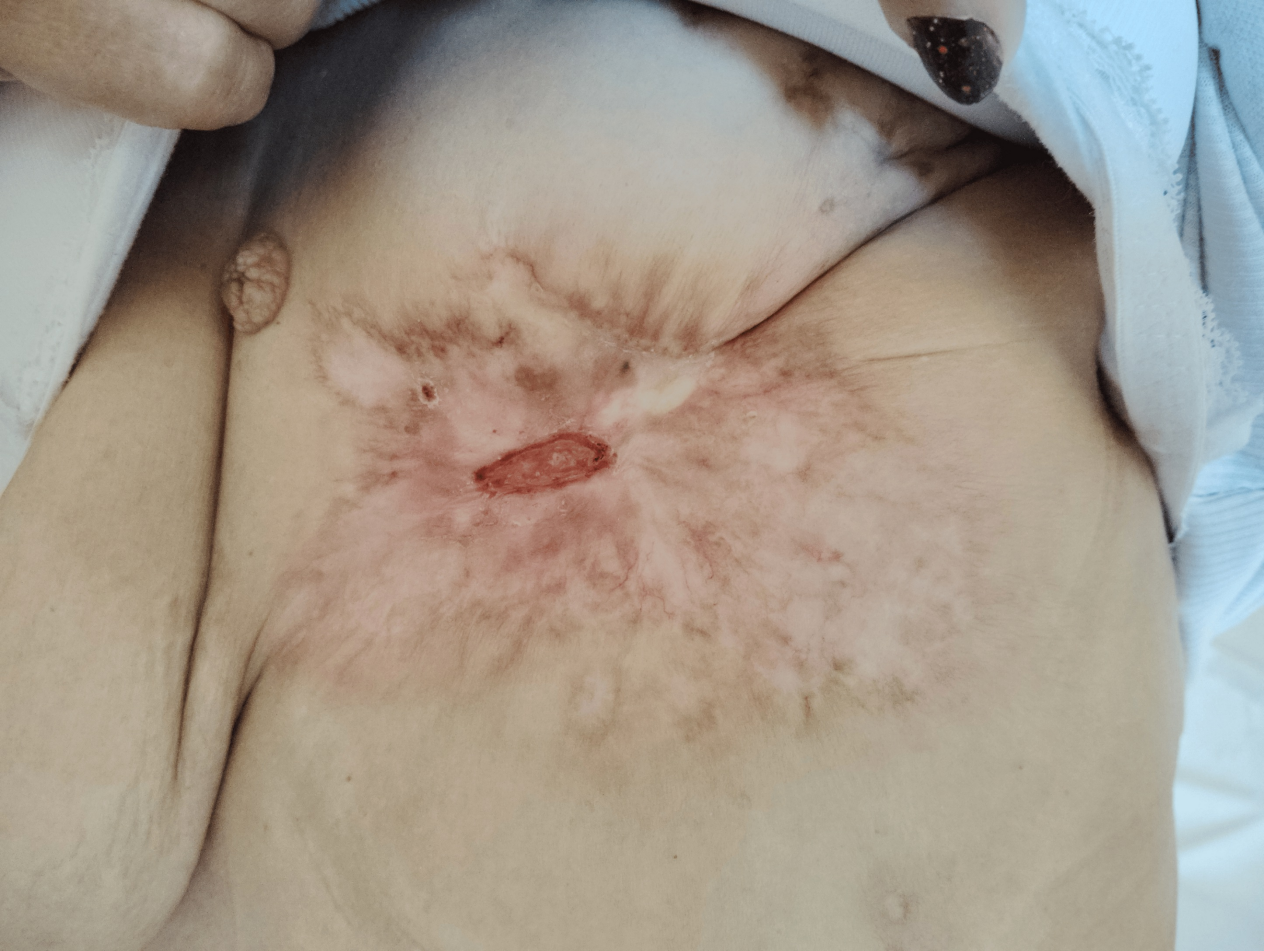
Nine cycle of letrozole with palbociclib

A restaging PET/CT scan conducted on August 5, 2024, revealed an almost complete response, with significant regression of skin lesions and control of the pain syndrome (Figure 8). Metabolic response assessment demonstrated a decrease in SUVmax from 4.8 to 1.9 in the cutaneous lesion and from 7.2 to 3.5 in bone metastases. No new lesions were detected, supporting the sustained effectiveness of the treatment.

**Figure 9:**
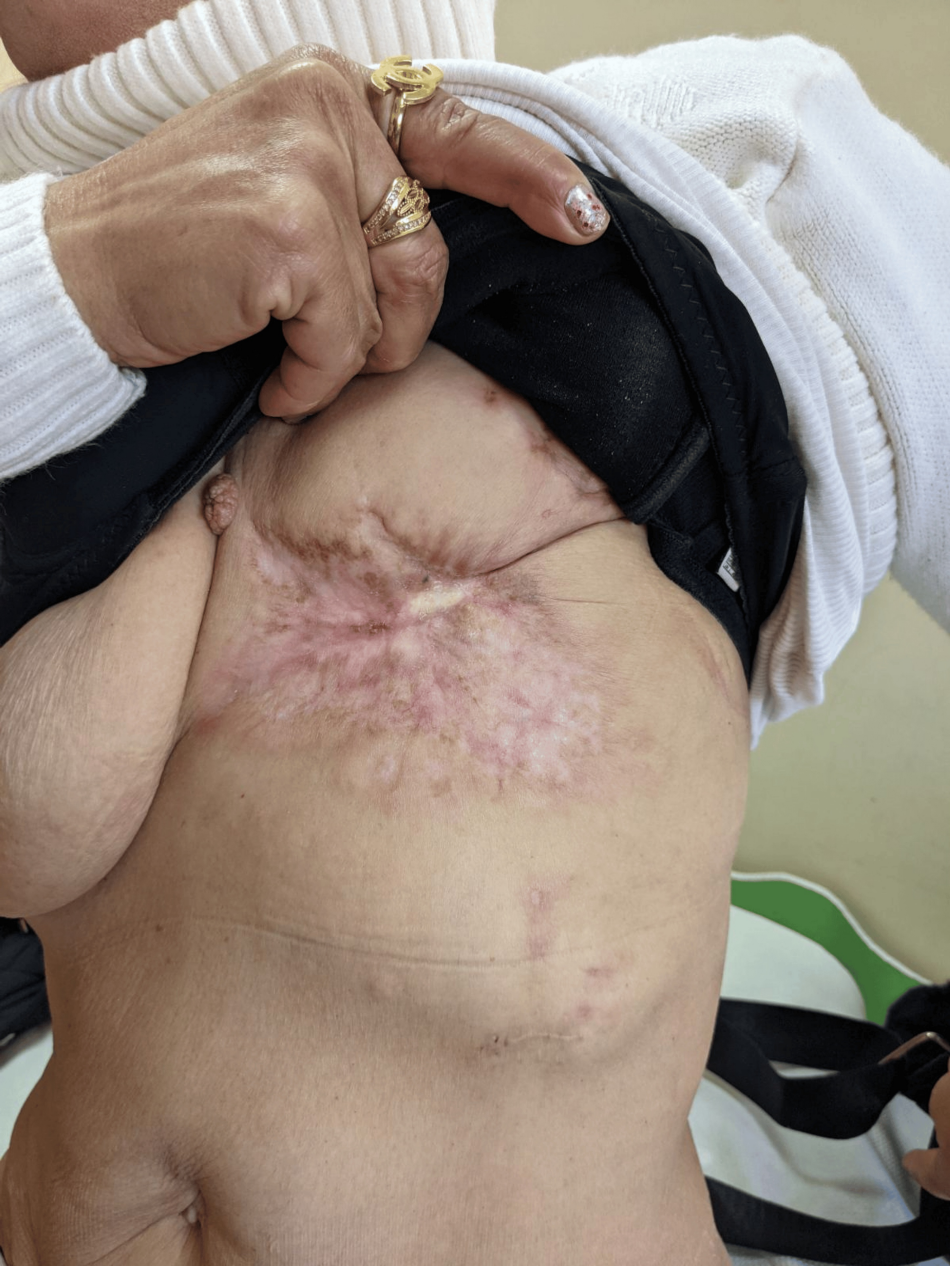
Tenth cycle of letrozole with palbociclib

Biochemical markers (Cancer antigen 15-3 and carcinoembryonic antigen) and clinical observations further supported the therapeutic success (Figure 9).

Tolerability and adverse events

The treatment was well tolerated. By December 2024, only mild neutropenia was observed due to palbociclib. There were no cases of hypocalcemia or osteonecrosis of the jaw from denosumab. Fentanyl was discontinued after effective pain control, with no significant side effects reported.

## Discussion

Based on the observed clinical outcomes and the absence of severe adverse events, the following discussion provides a broader context for the case.

The presented case highlights the effectiveness of combining letrozole, palbociclib, and denosumab in managing HR+, HER2-mBC with significant skin involvement, a condition often associated with poor prognosis and substantial impairment in quality of life. Cutaneous metastases in breast cancer are frequently refractory to treatment, leading to pain, ulceration, and increased risk of infection, which complicates disease management. Achieving durable disease control in this setting remains a clinical challenge.

These findings align with pivotal clinical trials, including PALOMA-2, MONALEESA-2, and MONARCH 3, which emphasize the survival benefits of integrating CDK4/6 inhibitors into standard endocrine therapy [7]. The introduction of CDK4/6 inhibitors has significantly improved PFS and OS in this patient population [8]. However, real-world data suggest that treatment adherence and toxicity management are critical factors influencing clinical outcomes outside of controlled trial settings [9].

Before the availability of CDK4/6 inhibitors, endocrine therapy was the primary treatment option, but its efficacy was often limited by acquired resistance [1,2]. Clinical trials have demonstrated that adding CDK4/6 inhibitors to endocrine therapy nearly doubles PFS compared to endocrine therapy alone, leading to prolonged disease control [3]. For instance, data from the MONALEESA-2 trial demonstrated that ribociclib combined with letrozole resulted in a median OS of 63.9 months compared to 51.4 months with letrozole alone [10]. Similarly, the MONARCH 2 trial revealed that abemaciclib combined with fulvestrant achieved a median OS of 46.7 months, significantly surpassing the 37.3 months observed with fulvestrant alone [11].

Despite these advances, several real-world considerations must be addressed. Treatment-related toxicities, particularly neutropenia, fatigue, and gastrointestinal side effects, frequently necessitate dose modifications or temporary treatment interruptions [12,13]. In clinical practice, achieving optimal therapeutic benefit while maintaining patient adherence can be challenging, especially in those with comorbidities [14]. Although real-world evidence supports the efficacy of CDK4/6 inhibitors, patient selection, and individualized dosing strategies remain essential for maximizing benefit and minimizing toxicity [15].

Although the patient achieved a near-complete response in skin lesions and significant pain control, the case underlines the importance of early intervention. Delayed diagnosis, as seen in this patient, remains a major challenge in clinical oncology and can limit treatment options and compromise long-term outcomes [16]. Furthermore, while bone-modifying agents such as denosumab are routinely used for skeletal protection in metastatic breast cancer, their potential role in modifying disease progression beyond bone metastases remains an area of investigation [17].

This case also underscores the tolerability of the combined regimen, with only mild neutropenia observed during therapy. However, it is important to recognize the limitations of single-case reports. Larger observational studies and prospective trials focusing on patients with cutaneous metastases are needed to validate these findings and optimize treatment sequencing strategies [18].

Future directions may include evaluating long-term outcomes and exploring biomarkers for predicting response to CDK4/6 inhibitors in similar clinical scenarios. Recent reviews and meta-analyses also highlight the long-term safety and evolving efficacy of CDK4/6 inhibitors in advanced breast cancer management, reinforcing their transformative potential in clinical oncology [19,20].

## Conclusions

This case demonstrates the successful disease control achieved with the combination of letrozole, palbociclib, and denosumab in a patient with HR+, HER2-negative mBC with cutaneous involvement. The regimen led to a near-complete response of skin lesions, significant symptom relief, and sustained disease stabilization without severe adverse events. Given the challenging prognosis associated with cutaneous metastases, achieving such a durable response highlights the efficacy of CDK4/6 inhibitors in complex clinical scenarios.

The absence of significant toxicity further supports the favorable safety profile of this regimen, allowing for long-term treatment continuity. This case reinforces the importance of early intervention and tailored therapeutic strategies, particularly in patients with uncommon metastatic patterns, to maximize clinical benefit and improve quality of life.
